# Tube Thoracostomy: Complications and Its Management

**DOI:** 10.1155/2012/256878

**Published:** 2011-10-16

**Authors:** Emeka B. Kesieme, Andrew Dongo, Ndubueze Ezemba, Eshiobo Irekpita, Nze Jebbin, Chinenye Kesieme

**Affiliations:** ^1^Department of Surgery, Irrua Specialist Teaching Hospital, PMB 8, Irrua, Edo State, Nigeria; ^2^National Cardiothoracic Center, University of Nigeria Teaching Hospital, Enugu, Nigeria; ^3^Department of Surgery, University of Port-Harcourt Teaching Hospital, PMB 6173, Port-Harcourt, Nigeria; ^4^Paediatric Intensive Care Unit, Irrua Specialist Teaching Hospital, PMB 8, Irrua, Edo State, Nigeria

## Abstract

*Background*. Tube thoracostomy is widely used throughout the medical, surgical, and critical care specialities. It is generally used to drain pleural collections either as elective or emergency. Complications resulting from tube thoracostomy can occasionally be life threatening. *Aim*. To present an update on the complications and management of complications of tube thoracostomy. *Methods*. A review of the publications obtained from Medline search, medical libraries, and Google on tube thoracostomy and its complications was done. *Results*. Tube thoracostomy is a common surgical procedure which can be performed by either the blunt dissection technique or the trocar technique. Complication rates are increased by the trocar technique. These complications have been broadly classified as either technical or infective. Technical causes include tube malposition, blocked drain, chest drain dislodgement, reexpansion pulmonary edema, subcutaneous emphysema, nerve injuries, cardiac and vascular injuries, oesophageal injuries, residual/postextubation pneumothorax, fistulae, tumor recurrence at insertion site, herniation through the site of thoracostomy, chylothorax, and cardiac dysrhythmias. Infective complications include empyema and surgical site infection. *Conclusion*. Tube thoracostomy, though commonly performed is not without risk. Blunt dissection technique has lower risk of complications and is hence recommended.

## 1. Introduction

Tube thoracostomy is the most commonly performed surgical procedure in thoracic surgery. As a life saving procedure, general surgeons, intensivists, emergency physicians, and respiratory physicians may at one time or the other be required to perform tube thoracostomy.

The first documented description of a closed tube drainage system for the drainage of empyema was by Hewett in 1867 [1]. However during the Second World War, the experience gained in military and civilian hospitals contributed to the development of tube thoracostomy in chest trauma management, and, at the time of the Vietnam war, it has become the standard of care for management of chest trauma [2]. In 1992, Lilienthal reported the postoperative use of chest tube following lung resection for suppurative diseases of the lung [3].

Tube thoracostomy is an invasive procedure and complications can result due to inadequate knowledge of thoracic anatomy or inadequate training and experience. These complications can simply be classified as technical or infective. Trocar technique is by far associated with a higher rate of complication [4, 5].

## 2. Methods

A literature review on tube thoracostomy was done from 1970 to date using manual library search, journal publications on the subject, and Medline. Full texts of the materials, including those of relevant references, were collected and studied. Information relating to the techniques, complications, and management of these complications were extracted from these materials.

## 3. Results

There are two principal methods of tube thoracostomy: the blunt dissection technique and the trocar technique. The trocar technique is associated with a higher rate of intrathoracic organ injury [4]. A combination of both trocar technique and blunt dissection technique has been described [4].

 Complications of tube thoracostomy can be classified as either technical or infective. Technical causes include tube malposition, blocked drain, chest drain dislodgement, reexpansion pulmonary edema, subcutaneous emphysema, nerve injuries, cardiac and vascular injuries, oesophageal injuries, residual/postextubation pneumothorax, fistulae, tumor recurrence at insertion site, herniation through the site, chylothorax, and cardiac dysrhythmias. Infective complications include empyema and surgical site infection including cellulitis and necrotizing fasciitis. 

## 4. Discussion

### 4.1. Applied Anatomy

A sound knowledge of the anatomy of the thorax is important to avoid some complications of tube thoracostomy.

**Figure 1:**
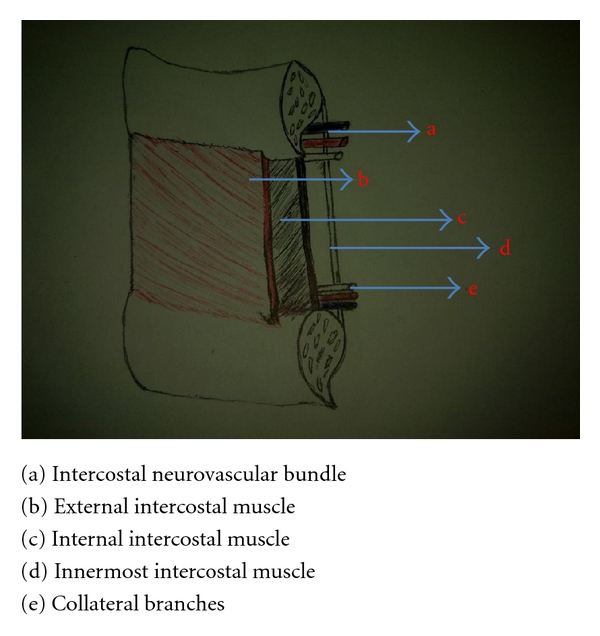
Anatomy of intercostal space.

The intercostal spaces are filled with intercostal muscles, with the vein, artery, and nerve lying in the costal groove along the inferior margin of the superior rib from above downwards and situated between the second and the third layer of muscles (Figure 1). To avoid the neurovascular bundle, it is normally advocated that the drain be located in the interspace just superior to the rib. However, puncture done as close as possible to the superior margin of the inferior rib may lead to laceration of the collateral intercostal artery [6]. Recent study has shown that the ideal spot should be 50–70% of the way down the interspace [7]. Injury to this neurovascular bundles remain possible complication of the procedure.

**Figure 2:**
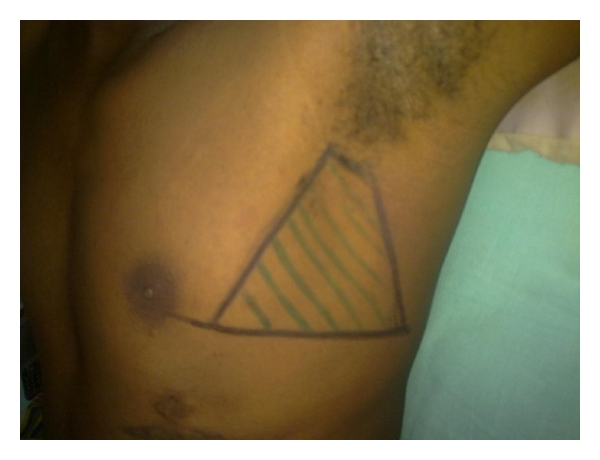
Triangle of safety.

British Thoracic Society (BTS) has recommended the triangle of safety as the site for insertion for intercostal drain [8]. This area is bordered by the anterior border of the latissimus dorsi, the lateral border of the pectoralis major muscle, a line superior to the horizontal level of the nipple, and an apex below the axilla (Figure 2). A survey of junior residents on the anatomical landmarks when inserting an intercostal drain revealed that 45% were placed outside the safe area of chest drain insertion with the most common error (20%) being a choice of insertion too low [9].

 The midaxillary line is the most commonly advocated position for tube thoracostomy; the innermost layer of intercostal muscle being poorly developed at this point, and comprising thin intracostals, which blend with the internal intercostal layer except where separated by neurovascular bundles [10]. A more anterior position will lead to injury to the muscles and breast tissue while a more posterior position is more uncomfortable and has risk of drain leakage. The long thoracic nerve lies behind the midaxillary line on the surface of serratus anterior and deep to the fascia and segmentally supplies this muscle.

In full expiration, the two domes rise as high as the 4th dorsal intervertebral space on the right and 5th space on the left; hence, when a chest tube is placed too low, there is a high probability of abdominal placement. Inferior placement of chest tubes will not only perforate the diaphragm but will also damage intra-abdominal organs. The same will also apply to other conditions that elevate the diaphragm, for example, late pregnancy, gross obesity, massive ascites, and intraabdominal tumours [10]. 

Each lung is invested by and enclosed in a serous pleural sac that consists of visceral and parietal pleura. Parietal pleura is subsequently divided into costal, diaphragmatic, cervical, and mediastinal pleura. The anatomical relationship to mediastinal structures explains injuries to these organs when a chest tube is inappropriately placed too far into the chest.

The right lung consists of three lobes (upper, middle and lower) separated by the horizontal and oblique fissures while the left consists of two lobes (upper and lower) separated by an oblique fissure. The position of the oblique fissure in either lung can be shown by a line drawn from the spinous process of the second thoracic vertebra around the side of the thorax to the sixth rib in the midclavicular line while the horizontal fissure runs at the level of the fourth costal cartilage and meets the oblique fissure in the midaxillary line. Chest tubes placed laterally in the fifth, sixth or seventh intercostal space will enter the chest near the oblique fissure and if directed centrally can enter the fissure [11]. Both the lung parenchyma and the fissures are thus potential sites of tube malposition.

## 5. Complications

### 5.1. Tube Malposition

Tube malposition is the commonest complication of tube thoracostomy [5, 12]. It is more common when tubes are inserted under suboptimal conditions and in urgent tube thoracostomy. Trocar technique of chest tube insertion has been shown to increase the risk of tube malposition compared with the blunt dissection techniques [5].

Complication rates of tube thoracostomy have been found to be higher in the critically ill patients with about 21% of tubes placed intrafissurally and 9% intraparenchymally [13].

Tube malposition has been defined by CT confirmation in 4 locations: intraparenchymal, fissural, extrathoracic, and angulation of the drain in the pleural space [4]. In this review, tube malposition will be classified as intraparenchymal tube placement, fissural tube placement, chest wall tube placement, mediastinal tube placement, and abdominal tube placement. 

#### 5.1.1. Intraparenchymal Tube Placement

Intraparenchymal chest tube placement occurs more likely in the presence of pleural adhesions or preexisting pulmonary disease [14]. It may be dramatic if there is associated injury to pulmonary vessels. However, clinical manifestation may be absent, and the only clue to the diagnosis of tube malposition may be inadequate drainage of air and fluid. The routine frontal and lateral radiographs taken after chest tube insertion may be unreliable in demonstrating the exact location of the tube. In contrast, chest computerized tomographic (CT) scan has been shown to be superior and more accurate than plain radiographs in assessing malpositioned chest tubes and also in providing additional valuable information with significant therapeutic impact [5, 12]. The drawback of computerized tomographic scan of chest in the developing countries is its unaffordability. 

#### 5.1.2. Fissural Tube Placement

The probability of interlobar malpositioning is significantly higher when using the lateral approach of tube thoracostomy as opposed to the anterior approach. Curtin et al. found no significant difference between intrafissural tube and those located elsewhere in the following outcome measures: duration of thoracostomy drainage, quantity of pleural fluid drained, need for further tubes, length of hospital stay, appearance on last chest radiograph before discharge and need for surgical intervention [15]. This finding contrasts the result of Stark et al. that showed that tubes that lie within the fissure correlated with a lengthy and complicated hospital course [12]. 

On anteroposterior chest radiograph, an interfissural tube is more likely to extend centrally or superiorly in a straight line or follow a gentle curve from its point of entry unlike the normally placed tube in the anterior or posterior pleural space, which will be angulated or will follow a sharp curve at its point of entry [11]. Lateral radiograph confirms the position. Malfunctioning interfissural tubes should be repositioned or replaced to improve function. 

#### 5.1.3. Chest Wall Tube Placement

Subcutaneous tube placement is a rare complication with reported incidence between 1–1.8% [16]. An unstable chest wall secondary to multiple rib fractures, haematoma, and hurried chest tube insertion was suspected to be the etiological factor in a case reported by Özpolat and Yazkan [17]. It can be identified clinically by tube malfunctioning and the lack of fluctuation of the fluid level in the drainage system and radiologically by subcutaneous position of the chest tube. This complication can be minimized by blunt dissection technique. Subcutaneous tube should be removed and replaced correctly into the pleural cavity.

#### 5.1.4. Mediastinal Tube Placement

Placing chest tubes far into the thorax can result in perforation of heart, injuries to large vessels, perforation of the oesophagus, and nerve injuries. Details of these injuries will be discussed separately under cardiovascular injuries, oesophageal perforation, and nerve injuries.

#### 5.1.5. Abdominal Placement

Triangle of safety has been advocated as the correct site for tube thoracostomy. Abdominal placement of tube usually occurs when tube thoracostomy is performed too low below this area. Injuries to the spleen, liver, and stomach have all been reported secondary to inadvertent passage of tube through the diaphragm [18]. Perforation of intra-abdominal viscera by chest tube is also possible in acquired diaphragmatic rupture with visceral herniation [19]. Injury to hollow viscus requires repair. The extent of surgery on a perforated solid viscus depends on the degree of injury. 

### 5.2. Blocked Drain

**Figure 3:**
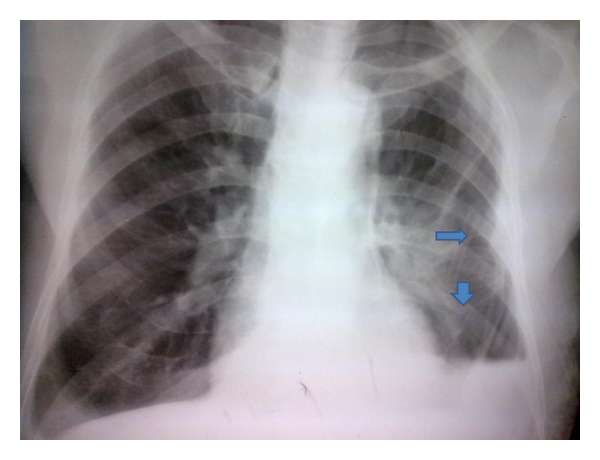
Kinking of chest drain.

Nonfunctional drain may be due to kinking (Figure 3), angulation, clot formation within the lumen or the presence of debris, or lung tissue. Smaller drains tend to kink or clot easier than larger drains especially when used in the setting of trauma [20]. A cardinal sign of blocked chest tube drain is failure of fluid within the tube to fluctuate with coughing or respiration. This ineffective drainage will result in undrained or unresolved pleural collection. Tension pneumothorax can also result in cases of ongoing air leak. Milking or stripping can be used to unblock semisolid contents, for example, blood clots or fibrin clots blocking the lumen of the tube. However, this is controversial and debatable as the negative pressure created may damage lung tissue. Chest drain should be unkinked in cases of kinking causing blockage.

### 5.3. Chest Drain Dislodgement

This can be partial or total. It can be prevented by meticulous care and good technique of drain anchorage. The use of mattress suture or a stay-in closure suture to secure the chest tube is recommended [8]. The use of purse-string suture in securing chest drain is complicated by poor cosmetic results and increased risk of skin necrosis. The seal provided by purse-string suture does not prevent air leaks [21]. The ideal suture to secure the tube should be strong and nonabsorbable, for example, “1” silk should include adequate skin and subcutaneous tissue to ensure it is secure [8]. A dislodged chest tube should be reintroduced with aseptic precaution through a new site.

### 5.4. Reexpansion Pulmonary Edema (REPE)

This is an uncommon but fatal complication that can occur following tube thoracostomy for pneumothorax or pleural effusion. Mortality rate of up to 20% has been reported [22]. 

Reexpansion pulmonary edema has also been reported following transthoracic endoscopic sympathectomy for primary hyperhidrosis [23], reexpansion of lung after decortications [24], reexpansion after excision of giant mediastinal tumour [25], and following puncture of a giant bulla [26]. It usually occurs on the side ipsilateral to the reexpanded lung though cases of reexpansion pulmonary edema occurring on the side contralateral to the reexpanded lung [27] and even bilateral reexpansion pulmonary edema have been reported [28].

The aetiology of REPE is unknown, but certain hypotheses have been suggested. The most important pathophysiological mechanism appears to be increased endothelial permeability and loss of integrity of the alveolar capillaries leading to exudation of protein-rich fluid. The factors responsible for this include.

- Lung collapse itself: Destruction of pulmonary microvascular endothelium occur, probably due to anoxic stress, mechanical stress exerted on the endothelium by blood cells, and changes in lymph flow.
- The mechanical stress to normal vessels during reexpansion
- Increase in oxygen-free radical and increase of activity of its scavenger, catalase, in a reexpanded lung [29]. Oxygen-free radical may injure capillary endothelium. This hypothesis is supported by the observation that inhalation of oxygen at FiO_2_ of 0.4 prevents pulmonary edema when lungs are reexpanded [30].
- Increase in leucocyte sequestration, interleukin-8 (1L-8), monocyte chemoattractant protein-1 (MCP-1) [31]. An increase in the level and activity of xanthine oxidase has also been reported [32].

This increase in IL-8 and monocyte chemoattractant protein (MCP) does not only occur in the affected lung, but also occurs to a lesser extent in the contralateral lung, partly explaining the phenomena of contralateral and bilateral reexpansion pulmonary edema [31, 33]. However, the main cause of contralateral REPE is thought to be secondary to the compression atelectasis of the contralateral lung associated with shift of mediastinum [34].

(e) Low levels of surfactants have been noted in chronically collapsed lung [35]. Decrease of alveolar surfactant activity is said to include pulmonary edema by drastically lowering the intrapleural pressure and further lowering the perivascular pressure of the pulmonary microvessels.

The risk factors for developing REPE in a patient include young age (<40 years), collapse of the affected lung for more than 3 days, large pneumothorax (>30% of single lung), application of significant negative pleural pressure suction and rapid lung reexpansion [22, 36, 37]. 

Clinical picture varies from asymptomatic radiologic findings to dramatic respiratory failure with circulatory shock. Patient usually becomes symptomatic within 2 hours after rapid lung reexpansion. There may be frothy sputum production associated with tachypnea, tachycardia, and cyanosis. Auscultation may reveal rales, and chest radiograph will reveal the presence of pulmonary infiltrates with ground glass appearance. 

Prophylactic measures include recognizing patients at high risk, leaving thoracostomy tubes initially off suction, preferring underwater seal drainage rather than negative pressure apparatus, and ensuring that fluid exceeding 1 L must not be removed rapidly if the pleural pressure is not being monitored. The goal should be to keep the pleural pressure above −20 cm H_2_O [38].

Treatment includes haemodynamic support (vigorous resuscitation and vasopressors), administration of supplemental oxygen, and, if need be, mechanical ventilation with positive end-expiratory pressure (PEEP). In cases of unilateral REPE, positioning the patient in lateral decubitus position with the affected side up will reduce intrapulmonary shunting and improve oxygenation [39].The use of NSAIDS is not evidence based. Diuretics should be avoided due to the hypovolaemic status.

### 5.5. Subcutaneous Emphysema

**Figure 4:**
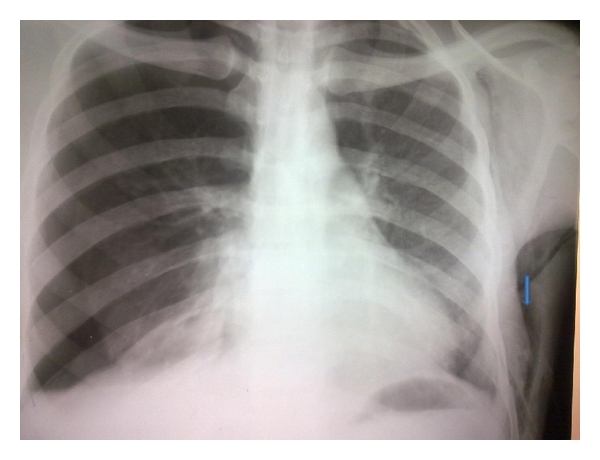
Subcutaneous emphysema complicating tube thoracostomy.

Development of subcutaneous emphysema is a known complication of tube thoracostomy. It usually presents as subcutaneous crepitation demonstrable clinically or as an occult radiologic finding (Figure 4). Extensive subcutaneous emphysema may present with extreme discomfort, disfigurement, anxiety, upper airway obstruction [40], and pacemaker dysfunction [41].

Subcutaneous emphysema following chest tube insertion is more commonly associated with trauma, bronchopleural fistula, large and bilateral pneumothoraces, and mechanical ventilation. There is an established association with prolonged drainage, poor tube placement, tube blockage, side port migration, and a greater number of chest tubes. It results in a longer length of hospital stay and increased mortality [42].

Subcutaneous emphysema resulting from chest tube insertion is usually minor and self-limiting. Other modalities that have been tried in managing extensive subcutaneous emphysema include infraclavicular blow-holes (incising the skin and subcutaneous fascia to allow air to escape) [43], insertion of fenestrated angiocatheter into the subcutaneous tissue [44], subcutaneous pigtail [45], or large bore drains [46]. These modalities are rarely necessary.

### 5.6. Nerve Injuries

#### 5.6.1. Horner's Syndrome

Horner's syndrome has been reported in the adult and paediatric populations [47, 48]. It is an oculosympathetic paresis, resulting from interruption of second-order preganglionic neurons and manifest as miosis, ptosis, hemifacial anhidrosis, and enophthalmos. Horner's syndrome results from direct pressure of the tip of the chest tube on the sympathetic chain in the medial portion of the apex. A thin endothoracic fascia separates the parietal pleura from the ganglion [49]. This complication is therefore avoided by not placing the tip of the tube close to the apex.

The malpositioned tube should be pulled 2-3 cm back as soon as possible after radiological confirmation. The resolution can be complete, partial, or absent depending on the degree of injury to the ganglia.

#### 5.6.2. Phrenic Nerve Injury

Diaphragmatic paralysis is an uncommon complication of tube thoracostomy, mostly reported in the paediatric population [50, 51]. The underlying aetiology is injury to the phrenic nerve secondary to tube malposition. Clinical suspicion should be confirmed by chest radiograph, fluoroscopy, nerve conduction studies, and magnetic resonance imaging (MRI). MRI may reveal haematoma in the region of the chest tube tip and phrenic nerve fibers [51]. Chiladiati sign occurring probably as a result of diaphragmatic paralysis has been reported by Gulati et al. [52].

Diaphragmatic paralysis following tube thoracotomy can be prevented by correct positioning of the chest tube tip at least 2 cm distant from the vertebrae [51]. Prompt recognition and correction of malpositioned tube or selection of softer chest tube is advised. Symptomatic cases with intractable respiratory distress should be managed by diaphragmatic plication.

#### 5.6.3. Injury to Long Thoracic Nerve

Injury to long thoracic nerve of Bell causing winging of the scapula has been reported as a possible complication of tube thoracostomy [53]. Physiotherapy is the hallmark of treatment. Recovery of full muscle strength and complete recovery is possible after 6 months of regular physiotherapy.

#### 5.6.4. Ulnar Neuropathy

This is a rare, though significant, complication associated with tube thoracostomy. Repositioning of tube leads to significant improvement. Management of persistent symptoms is expectant, with early upper extremity range of motion and strength exercise [54].

### 5.7. Cardiac and Vascular Injuries

#### 5.7.1. Cardiac Injuries

Perforation of the heart is a rare catastrophic complication of tube thoracostomy. Injuries with perforation of the (R) atrium [55], (L) atrium [56], (R) ventricle [57], and the (L) ventricle [58] have all been reported. Predisposing factors in patients reported were thoracic deformities, enlarged cardiac chambers, trauma, and emergency respiratory conditions.

Miesel et al. reported perforation of the (R) atrium using trocar type thoracotomy technique in a patient with kyphoscoliosis [55]. Perforation of the (R) ventricle has occurred due to poor knowledge of the anatomy of the postpneumonectomy space by operating physician [57].

Perforation of the heart leads to immediate return and continuous stream of blood emerging from the chest tube leading to marked hypotension, haemorrhagic shock, and death. Prompt intervention is required. Perforation should be repaired by polypropylene suture via a thoracotomy.

#### 5.7.2. Injury to the Pulmonary Artery

Injuries to the pulmonary artery and pulmonary artery pseudoaneurysm are rare serious complications of chest tube insertion [59–62].

These injuries occurred mainly with the use of Trocar method of chest tube insertion; however, this complication has occurred following blunt dissection technique [60]. In all patients reported, there was background dense pleural adhesion in the pleural space [59, 60] or postpneumonectomy states [61]. Dense pleural adhesion prevents the normal entry into the pleural space leading to lung penetration and subsequent perforation of the pulmonary artery. Diagnosis is made by the immediate return of frank blood through the tube and subsequent hypotension. 

Definitive management is surgical [63] although nonoperative management has been reported by Sundaramurthy et al. [60]. Nonoperative management involves the occlusion of the pulmonary artery perforation by clamped chest tube and the formation of clots in the tract as the tube is gradually withdrawn. The concern with this approach is the unpredictability of haemostasis and the likelihood of thrombosis of the pulmonary artery with clot propagation [64]. The aim of surgery is to repair the pulmonary artery. If this is not feasible, then a pneumonectomy may be performed. Dense pleural adhesions usually make surgery technically difficult.

#### 5.7.3. Occlusion of Subclavian Artery

Subclavian artery obstruction following tube thoracostomy is rare. Fowler reported subclavian artery occlusion in a premature baby who had (R) closed tube thoracostomy drainage after thoracotomy for repair of tracheoesophageal fistula with oesophageal atresia [65]. A case of an obstruction to subclavian artery which was not physiologically significant was reported by Moskal et al. [66]. Both cases were treated by repositioning of chest tube.

#### 5.7.4. Intercostal Artery Injury and Chest Wall Arteriovenous Fistula

This is a cause of haemorrhage during the insertion of chest tube. Intercostal arteries may bleed profusely when traumatized. Dissection during tube insertion should be done above the superior border of the rib to avoid the neurovascular bundles on the groove located on the inferior aspect. The safest zone to perform tube thoracostomy should be between 50–70% of the way down an interspace to avoid the variably positioned superior intercostal neurovascular bundle and the inferior collateral artery [7]. Systemic arteriovenous fistula (SAVF) involving an intercostal artery and subcutaneous vein can result after tube thoracostomy [67]. The clinical manifestations of a traumatic SAVF may be immediate or delayed, ranging from 1 week to 12 years. The classical physical signs are pulsatile mass, palpable thrill, and machinery murmur. Chest radiography may reveal bone density with bone erosion. However, the gold standard for investigation is selective angiography. Modalities of treatment include surgery and more recently, transcatheter embolization.

### 5.8. Residual/Postextubation Pneumothorax

This is avoided during the removal of chest tube by maintaining a sustained valsalva manoeuvre to forcibly inflate the lung against the chest wall with breathing suspended until the purse string is tied. Residual pneumothorax may be secondary to persistent air leak from underlying pathology. Repeat tube thoracostomy is indicated if pneumothorax is significant or if it is secondary to persistent air leak. However, in persistent air leak, chest tube should not be removed prematurely. Small residual/postextubation pneumothorax requires no intervention.

### 5.9. Esophageal Perforation

Esophageal perforation following closed tube thoracostomy drainage has been reported both in the normal esophagus and at the site of esophageal anastomosis/myotomy following repair of esophageal atresia [68, 69].

Drainage of enteric contents is pathognomonic of this condition. Diagnosis is confirmed by contrast studies. The goal of management is to eliminate the source of soilage and to ensure adequate nutrition. 

Conservative management with feeding gastrostomy was used to manage a reported case [70], but surgery is the mainstay of treatment. Options of surgery include primary repair with or without buttressing of suture lines, muscle flap closure, exclusion and diversion, drainage, and resection. This complication can be avoided by early recognition and repositioning of chest tube.

### 5.10. Fistula

#### 5.10.1. Acquired Bronchocutaneous Fistula

Bronchocutaneous fistula is a pathologic communication between the bronchus, pleural space, and subcutaneous tissue. Acquired bronchocutaneous fistula has been reported as a complication of tube thoracostomy [71]. Because of constant air leak, this complication needs to be treated immediately to prevent devastating pulmonary infection. Treatment options include endoscopic repair, parietal pleurectomy, and pleurodesis.

#### 5.10.2. Pleurocutaneous Fistula

Pleurocutaneous fistula is defined as a pathologic communication between the pleural space and the subcutaneous tissues. Pleurocutaneous fistula secondary to tube thoracostomy has been reported in few studies [72]. Patients with pleurocutaneous fistula exhibit no physical signs. Computerized tomographic scan of the chest is usually the mainstay of diagnosis; however, chest ultrasonography has been found to be useful both as a tool for making diagnosis and following up these patients. Treatment is directed both at the casual agent and predisposing factor.

### 5.11. Tumor Recurrence at Insertion Sites

Tumour recurrence is possible at thoracostomy tube insertion sites. This may be a manifestation of a local spread or distant metastasis, and this complication is more related to the postsurgical procedure rather than percutaneous chest tube placement itself. Hayes-Jordan et al. reported 2 cases of tumor recurrence at thoracostomy tube insertion sites after intraoperative gross spillage of pleuropulmonary blastoma and malignant epithelial thymoma [73]. Caution must be exercised to avoid spillage of tumours at the time of resection especially in tumours that are relatively resistant to chemotherapy and radiotherapy. Treatment will depend on the response of the primary tumour to the modalities of surgery, chemotherapy, or radiotherapy.

### 5.12. Cardiac Dysrhythmia

Arrthymias may rarely complicate chest tube insertion [74–76]. These may result from mechanical stimulation of the heart or its covering, the pericardium or due to irritation of the vagus nerve. Sudden death due to a profound unresponsive bradycardia has been documented following tube thoracostomy that resulted in haemorrhage and irritation of the vagus nerve [74].

In patients that presented with atrial fibrillation following chest tube insertion, factors that suggested that the tube as the culprit included the close association between the time the chest tube was inserted and the onset of arrhythmias, cessation of arrhythmias following tube withdrawal, no further occurrence of arrhythmias, location of kinked chest tube by chest radiograph, and proximity to the right atrium [75, 76]. Antiarrhythmic agents are not beneficial in this condition. Chest tube should be withdrawn and the effect on arrhythmias monitored.

### 5.13. Herniation of a Lung Bulla through Insertion Site

A previous thoracostomy site may serve as a weak point on the chest wall allowing herniation. Three cases of herniation of emphysematous bullae through a previous tube thoracostomy site have been reported [77–79]. The reported cases were mainly managed by surgical excision through a thoracotomy though there is room for conservative management of herniated bullae.

### 5.14. Chylothorax

Chylothorax has been reported as a late complication of traumatic chest tube insertion with injury to the thoracic duct [80]. This complication should be included in the differential diagnoses of patients presenting with chylothorax after tube thoracostomy. Line of management may be conservative (limited oral intake and supplementation with medium chain triglycerides (MCTs), which directly enter the portal system) or surgical.

### 5.15. Infectious Complication

Closed tube thoracostomy is classified as “clean contaminated” and hence risk of infection of wound is 7.7%. Studies of empyema secondary to tube thoracostomy have reported complication rates as low as 1% and as high as 25% [81, 82]. Studies have shown that the rate of empyema is higher when pleural effusion was present before tube thoracostomy [81]. The presence of pleural effusion allows nosocomial colonization from respiratory tract leading to subsequent empyema.

The usefulness of prophylactic antibiotics following tube thoracostomy has remained controversial. Prophylactic antibiotics have been found unnecessary in patients with primary spontaneous pneumothorax who require closed tube thoracostomy [83]. This has been shown not only to be very cost effective but also prevent complications from antibiotics abuse.

Concerning tube thoracostomy following chest trauma, there is insufficient evidence to support the blanket use of prophylactic antibiotics for all patients though antibiotics prophylaxis is appropriate for those at an increased risk of developing infectious complications

Many studies have revealed significant reduction in empyema and pneumonia in patients who sustained chest trauma and require tube thoracostomy and were placed on prophylactic antibiotics compared with placebo [84, 85]. However, based on flawed methodology, these conclusions cannot be supported. Empyema occurs more frequently after penetrating chest trauma than blunt chest trauma as penetrating injuries allow direct entry of microorganisms into the pleural space. 

Surgical site infection can range from cellulitis to necrotizing soft tissue infection. Tube thoracostomy drainage for empyema thoracis has a higher probability of giving rise to necrotizing soft tissue infection. Prevention of wound site infection is by adequate skin preparation. Cellulitis usually responds to antibiotics.

Necrotizing fasciitis after tube thoracostomy has been reported complicating empyema thoracis [86], secondary spontaneous pneumothorax for tuberculosis [87] and in patient with Werdnig-Hoffman disease [88].

Infection usually begins as an area of redness that will later give rise to dusky and purplish skin, signs of tissue necrosis, putrid discharge, bullae, severe pain, and subcutaneous emphysema with systemic features. Chest radiograph will reveal the presence of gas in the subcutaneous plane. Line of management involves antibiotics, aggressive surgical debridement, and repeat debridement as the case may be. Once infection is controlled, skin grafting is done. Free flap is seldom required but must be considered when treating more complex defects. Microsurgical reconstruction with latissimus dorsi free flap has been used for pleural reconstruction and wall stabilization [89]. The use of hyperbaric oxygen is being encouraged as it has a bacteriocidal effect, improve polymorphonuclear function, and enhance wound healing.

## 6. Conclusion

Tube thoracostomy is not without risk. Blunt dissection technique has lower risk of complications and is hence recommended. It is important to keep to the triangle of safety to limit these errors. Most of these complications are preventable and when they occur, they must be adequately and correctly managed.

## References

[1] Hewett FC (1876). Thoracentesis: the plan of continuous aspiration. *The British Medical Journal*.

[2] Monaghan SF, Swan KG (2008). Tube thoracostomy: the struggle to the ‘standard of care’. *Annals of Thoracic Surgery*.

[3] Lilienthal H (1922). Resection of the lung for suppurative infections with a report based on 31 operative cases in which resection was done or intended. *Annals of Surgery*.

[4] Dural K, Gulbahar G, Kocer B, Sakinci U (2010). A novel and safe technique in closed tube thoracostomy. *Journal of Cardiothoracic Surgery*.

[5] Baldt MM, Bankier AA, Germann PS, Pöschl GP, Skrbensky GT, Herold CJ (1995). Complications after emergency tube thoracostomy: assessment with CT. *Radiology*.

[6] Da Rocha RP, Vengjer A, Blanco A, de Carvalho PT, Mongon ML, Fernandes GJ (2002). Size of the collateral intercostal artery in adults: anatomical considerations in relation to thoracocentesis and thoracoscopy. *Surgical and Radiologic Anatomy*.

[7] Wraight WM, Tweedie DJ, Parkin IG (2005). Neurovascular anatomy and variation in the fourth, fifth, and sixth intercostal spaces in the mid-axillary line: a cadaveric study in respect of chest drain insertion. *Clinical Anatomy*.

[8] Laws D, Neville E, Duffy J (2003). BTS guidelines for the insertion of a chest drain. *Thorax*.

[9] Griffiths JR, Roberts N (2005). Do junior doctors know where to insert chest drains safely?. *Postgraduate Medical Journal*.

[10] Ellis H (2007). The applied anatomy of chest drain insertion. *The British Journal of Hospital Medicine*.

[11] Webb WR, LaBerge JM (1984). Radiographic recognition of chest tube malposition in the major fissure. *Chest*.

[12] Stark DD, Federle MP, Goodman PC (1983). CT and radiographic assessment of tube thoracostomy. *The American Journal of Roentgenology*.

[13] Remérand F, Luce V, Badachi Y, Lu Q, Bouhemad B, Rouby JJ (2007). Incidence of chest tube malposition in the critically ill: a prospective computed tomography study. *Anesthesiology*.

[14] Fraser RS (1988). Lung perforation complicating tube thoracostomy: pathologic description of three cases. *Human Pathology*.

[15] Curtin JJ, Goodman LR, Quebbeman EJ, Haasler GB (1994). Thoracostomy tubes after acute chest injury: relationship between location in a pleural fissure and function. *The American Journal of Roentgenology*.

[16] Bergaminelli C, De Angelis P, Gauthier P, Salzano A, Vecchio G (1999). Thoracic drainage in trauma emergencies. *Minerva Chirurgica*.

[17] Özpolat B, Yazkan R (2007). Ectopic chest tube insertion to thoracic wall. *Turkish Journal of Geriatrics *.

[18] Millikan JS, Moore EF, Steiner E, Aragon GE, Van Way CW (1980). Complications of tube thoracostomy for acute trauma. *The American Journal of Surgery*.

[19] Içöz G, Kara E, Ilkgül O, Yetgin S, Tunçyürek P, Korkut MA (2003). Perforation of the stomach due to chest tube complication in a patient with iatrogenic diaphragmatic rupture. *Acta Chirurgica Belgica*.

[20] Collop NA, Kim S, Sahn SA (1997). Analysis of tube thoracostomy performed by pulmonologists at a teaching hospital. *Chest*.

[21] Tang ATM, Velissaris TJ, Weeden DF (2002). An evidence-based approach to drainage of the pleural cavity: evaluation of best practice. *Journal of Evaluation in Clinical Practice*.

[22] Mahfood S, Hix WR, Aaron BL, Blaes P, Watson DC (1988). Reexpansion pulmonary edema. *Annals of Thoracic Surgery*.

[23] Lin TS, Wang NP, Huang LC (2001). Pitfalls and complication avoidance associated with transthoracic endoscopic sympathectomy for primary hyperhidrosis (analysis of 2200 cases). *International Journal of Surgical Investigation*.

[24] Yamanaka A, Hirai T, Ohtake Y, Watanabe M, Nakamura K, Tanabe T (1998). Surgery for thoracic empyema concurrent with rupture of lung abscesses in a child. *Journal of Pediatric Surgery*.

[25] Matsumiya N, Dohi S, Timura T, Naito H (1991). Reexpansion pulmonary edema after mediastinal tumor excision. *Anesthesia & Analgesia*.

[26] Fukuda T, Okutani R, Kono K, Ishida H, Yamanaka N, Okamoto E (1989). A case of reexpansion pulmonary edema during fenestration of giant hepatic cyst. *Masui*.

[27] Steckel RJ (1973). Unilateral pulmonary edema after pneumothorax. *The New England Journal of Medicine*.

[28] Ragozzino MW, Greene R (1991). Bilateral reexpansion pulmonary edema following unilateral pleurocentesis. *Chest*.

[29] Jackson RM, Russell WJ, Veal CF (1992). Endogenous and exogenous catalase in reoxygenation lung injury. *Journal of Applied Physiology*.

[30] Pavlin DJ, Nessly ML, Cheney FW (1987). Hemodynamic effects of rapidly evacuating prolonged pneumothorax in rabbits. *Journal of Applied Physiology*.

[31] Sakao Y, Kajikawa O, Martin TR (2001). Association of IL-8 and MCP-1 with the development of reexpansion pulmonary edema in rabbits. *Annals of Thoracic Surgery*.

[32] Saito S, Ogawa JI, Minamiya Y (2005). Pulmonary reexpansion causes xanthine oxidase-induced apoptosis in rat lung. *The American Journal of Physiology—Lung Cellular and Molecular Physiology*.

[33] Nakamura M, Fujishima S, Sawafuji M (2000). Importance of interleukin-8 in the development of reexpansion lung injury in rabbits. *The American Journal of Respiratory and Critical Care Medicine*.

[34] Sohara Y (2008). Reexpansion pulmonary edema. *Annals of Thoracic and Cardiovascular Surgery*.

[35] Katz S, Knight R (1976). Reexpansion pulmonary edema. *The American Family Physician*.

[36] Matsuura Y, Nomimura T, Murakami H, Matsushima T, Kakehashi M, Kajihara H (1991). Clinical analysis of reexpansion pulmonary edema. *Chest*.

[37] Miller WC, Toon R, Palat H, Lacroix J (1973). Experimental pulmonary edema following re-expansion of pneumothorax. *The American Review of Respiratory Disease*.

[38] Light RW, Light RW (2003). Thoracentesis and pleural biopsy. *Pleural Disease*.

[39] Trachiotis GD, Vricella LA, Aaron BL, Hix WR (1997). Reexpansion pulmonary edema: updated in 1997. *Annals of Thoracic Surgery*.

[40] Gibney RTN, Finnegan B, FitzGerald MX, Lynch V (1984). Upper airway obstruction caused by massive subcutaneous emphysema. *Intensive Care Medicine*.

[41] Giroud D, Goy JJ (1990). Pacemaker malfunction due to subcutaneous emphysema. *International Journal of Cardiology*.

[42] Jones PM, Hewer RD, Wolfenden HD, Thomas PS (2001). Subcutaneous emphysema associated with chest tube drainage. *Respirology*.

[43] Herlan DB, Landreneau RJ, Ferson PF (1992). Massive spontaneous subcutaneous emphysema. Acute management with infraclavicular ‘blow holes’. *Chest*.

[44] Perkins LA, Jones SF (2007). Resolution of subcutaneous emphysema with placement of subcutaneous fenestrated angiocatheter. *Respiratory Medicine Extra*.

[45] Crouch JD, Keagy BA, Delany DJ (1987). ‘Pigtail’ catheter drainage in thoracic surgery. *The American Review of Respiratory Disease*.

[46] Kelly MC, McGuigan JA, Allen RW (1995). Relief of tension subcutaneous emphysema using a large bore subcutaneous drain. *Anaesthesia*.

[47] Kaya SO, Liman ST, Bir LS, Yuncu G, Erbay HR, Unsal S (2003). Horner’s syndrome as a complication in thoracic surgical practice. *European Journal of Cardio-Thoracic Surgery*.

[48] Özel SK, Kazez A (2004). Horner’s syndrome secondary to tube thoracostomy. *Turkish Journal of Pediatrics*.

[49] Fleishman JA, Bullock JD, Rosset JS, Beck RW (1983). Iatrogenic Horner’s syndrome secondary to chest tube thoracostomy. *Journal of Clinical Neuro-Ophthalmology*.

[50] Hwang MS, Chu JJ, Su WJ (2005). Diaphragmatic paralysis caused by malposition of chest tube placement after pediatric cardiac surgery. *International Journal of Cardiology*.

[51] Nahum E, Ben-Ari J, Schonfeld T, Horev G (2001). Acute diaphragmatic paralysis caused by chest-tube trauma to phrenic nerve. *Pediatric Radiology*.

[52] Gulati MS, Wafula J, Aggarwal S (2008). Chilaiditi’s sign possibly associated with malposition of chest tube placement. *Journal of Postgraduate Medicine*.

[53] Hassan WU, Keaney NP (1995). Winging of the scapula: an unusual complication of chest tube placement. *Journal of Accident and Emergency Medicine*.

[54] Rosing JH, Lance S, Wong MS Ulnar neuropathy after tube thoracostomy for pneumothorax.

[55] Meisel S, Ram Z, Priel I, Nass D, Lieberman P (1990). Another complication of thoracostomy—perforation of the right atrium. *Chest*.

[56] Kerger H, Blaettner T, Froehlich C (2007). Perforation of the left atrium by a chest tube in a patient with cardiomegaly: management of a rare, but life-threatening complication. *Resuscitation*.

[57] Kopec SE, Conlan AA, Irwin RS (1998). Perforation of the right ventricle: a complication of blind placement of a chest tube into the postpneumonectomy space. *Chest*.

[58] Abad C, Padrón A (2002). Accidental perforation of the left ventricle with a chest drain tube. *Texas Heart Institute Journal*.

[59] Takanami I (2005). Pulmonary artery perforation by a tube thoracostomy. *Interactive Cardiovascular and Thoracic Surgery*.

[60] Sundaramurthy SR, Moshinsky RA, Smith JA (2009). Non-operative management of tube thoracostomy induced pulmonary artery injury. *Interactive Cardiovascular and Thoracic Surgery*.

[61] Klalingen KW, Stam J, Rauwerda J (1991). Complications of thoracostomy. *Chest*.

[62] Podbielski FJ, Wiesman IM, Yaghmai B, Owens CA, Benedetti E, Massad MG (1997). Pulmonary artery pseudoaneurysm after tube thoracostomy. *Annals of Thoracic Surgery*.

[63] Kao CL, Lu MS, Chang JP (2007). Successful management of pulmonary artery perforation after chest tube insertion. *Journal of Trauma*.

[64] Edwin F (2009). eComment: management options of tube thoracostomy-induced pulmonary artery injury. *Interactive Cardiovascular and Thoracic Surgery*.

[65] Fowler CL (1995). Subclavian artery compression from a chest tube after thoracotomy in a premature infant. *Pediatric Radiology*.

[66] Moskal TL, Liscum KR, Mattox KL (1997). Subclavian artery obstruction by tube thoracostomy. *Journal of Trauma*.

[67] Coulter TD, Maurer JR, Miller MT, Mehta AC (1999). Chest wall arteriovenous fistula: an unusual complication after chest tube placement. *Annals of Thoracic Surgery*.

[68] Shapira OM, Aldea GS, Kupferschmid J, Shemin RI (1993). Delayed perforation of the esophagus by a closed thoracostomy tube. *Chest*.

[69] Johnson JF, Wright DR (1990). Chest tube perforation of esophagus following repair of esophageal atresia. *Journal of Pediatric Surgery*.

[70] Johnson AO, Grillo IA, Adebonojo SA (1980). Esophagopleural fistula: a complication of chest intubation for empyema. *Journal of the National Medical Association*.

[71] John SK, Jacob S, Piskorowski T (2005). Bronchocutaneous fistula after chest-tube placement: a rare complication of tube thoracostomy. *Heart and Lung*.

[72] Lin MT, Shih JY, Lee YC, Yang PC (2008). Pleurocutaneous fistula after tube thoracostomy: sonographic findings. *Journal of Clinical Ultrasound*.

[73] Hayes-Jordan AA, Daw NC, Furman WL, Hoffer FA, Shochat SJ (2004). Tumor recurrence at thoracostomy tube insertion sites: a report of two pediatric cases. *Journal of Pediatric Surgery*.

[74] Ward EW, Hughes TE (1994). Sudden death following chest tube insertion: an unusual case of vagus nerve irritation. *Journal of Trauma*.

[75] Barak M, Iaroshevski D, Ziser A (2003). Rapid atrial fibrillation following tube thoracostomy insertion. *European Journal of Cardio-Thoracic Surgery*.

[76] Hsu KF, Wang HB, Hsieh CB (2010). Refractory atrial fibrillation following tube thoracostomy. *Canadian Medical Association Journal*.

[77] Konecny JA, Grosso MA, Fernandez J, Murphy D, McGrath LB (1999). Images in cardiothoracic surgery. Herniation of emphysematous bulla through a chest tube site. *Annals of Thoracic Surgery*.

[78] Rathinam S, Collins FJ (2003). Bullous herniation of the lung through an intercostal drain site. *European Journal of Cardio-Thoracic Surgery*.

[79] Okur E, Tezel C, Baysungur V, Halezeroglu S (2008). Extrathoracic herniation of a lung bulla through a tube thoracostomy site. *Interactive Cardiovascular and Thoracic Surgery*.

[80] Limsukon A, Yick D, Kamangar N (2008). Chylothorax: a rare complication of tube thoracostomy. *Journal of Emergency Medicine*.

[81] Chan L, Reilly KM, Henderson C, Kahn F, Salluzzo RF (1997). Complication rates of tube thoracostomy. *The American Journal of Emergency Medicine*.

[82] Helling TS, Gyles NR, Eisenstein CL, Soracco CA (1989). Complications following blunt and penetrating injuries in 216 victims of chest trauma requiring tube thoracostomy. *Journal of Trauma*.

[83] Olgac G, Aydogmus U, Mulazimoglu L, Kutlu CA (2006). Antibiotics are not needed during tube thoracostomy for spontaneous pneumothorax: an observational case study. *Journal of Cardiothoracic Surgery*.

[84] Maxwell RA, Campbell DJ, Fabian TC (2004). Use of presumptive antibiotics following tube thoracostomy for traumatic hemopneumothorax in the prevention of empyema and pneumonia—a multi-center trial. *Journal of Trauma*.

[85] Gonzalez RP, Holevar MR (1998). Role of prophylactic antibiotics for tube thoracostomy in chest trauma. *The American Surgeon*.

[86] Kalkat MS, Rajesh PB, Hendrickse C (2003). Necrotizing fasciitis of chest wall complicating empyema thoracis. *Interactive Cardiovascular and Thoracic Surgery*.

[87] Hsu SP, Wang HC, Huang IT, Chu KA, Chang HC (2006). Tube thoracostomy-related necrotizing fasciitis: a case report. *Kaohsiung Journal of Medical Sciences*.

[88] Freixinet J, Rodríguez P, Santana N, Hussein M, Cruz F, Rodríguez de Castro F (2001). Necrotizing fasciitis of thoracic wall complicating chest tube drainage. *Asian Cardiovascular and Thoracic Annals*.

[89] Barbosa RF, Pinho CJ, Costa-Ferreira A, Cardoso A, Reis JC, Amarante JM (2006). Microsurgical reconstruction of chest wall defect after necrotizing fasciitis. *Microsurgery*.

